# Identification and characterization of the novel colonization factor CS30 based on whole genome sequencing in enterotoxigenic *Escherichia coli* (ETEC)

**DOI:** 10.1038/s41598-017-12743-3

**Published:** 2017-10-02

**Authors:** Astrid von Mentzer, Joshua Tobias, Gudrun Wiklund, Stefan Nordqvist, Martin Aslett, Gordon Dougan, Åsa Sjöling, Ann-Mari Svennerholm

**Affiliations:** 1 Department of Microbiology and Immunology, Sahlgrenska Academy, University of Gothenburg, Gothenburg, Sweden; 2 Pathogen Genomics, The Wellcome Trust Sanger Institute, Hinxton, Cambridge United Kingdom; 3 Microbial Pathogenesis Group, The Wellcome Trust Sanger Institute, Hinxton, Cambridge United Kingdom; 4 Department of Microbiology, Tumor and Cellbiology, Karolinska Institutet, Stockholm, Sweden

## Abstract

The ability to colonize the small intestine is essential for enterotoxigenic *Escherichia coli* (ETEC) to cause diarrhea. Although 22 antigenically different colonization factors (CFs) have been identified and characterized in ETEC at least 30% of clinical ETEC isolates lack known CFs. Ninety-four whole genome sequenced “CF negative” isolates were searched for novel CFs using a reverse genetics approach followed by phenotypic analyses. We identified a novel CF, CS30, encoded by a set of seven genes, *csmA-G*, related to the human CF operon CS18 and the porcine CF operon 987P (F6). CS30 was shown to be thermo-regulated, expressed at 37 °C, but not at 20 °C, by SDS-page and mass spectrometry analyses as well as electron microscopy imaging. Bacteria expressing CS30 were also shown to bind to differentiated human intestinal Caco-2 cells. The genes encoding CS30 were located on a plasmid (E873p3) together with the genes encoding LT and STp. PCR screening of ETEC isolates revealed that 8.6% (n = 13) of “CF negative” (n = 152) and 19.4% (n = 13) of “CF negative” LT + STp (n = 67) expressing isolates analyzed harbored CS30. Hence, we conclude that CS30 is common among “CF negative” LT + STp isolates and is associated with ETEC that cause diarrhea.

## Introduction

Enterotoxigenic *Escherichia coli* (ETEC) bacteria are highly diverse pathogens that harbor several virulence factors that play a role in causing diarrhea in infected individuals. ETEC infection is the most common cause of bacterial diarrhea in children in low and middle-income countries and in travelers to endemic areas^1^. ETEC are defined by the ability to produce a heat-labile toxin (LT) and/or heat-stable toxin (ST, including two subtypes STh and STp)^2^. STh is produced by ETEC isolated from humans whereas STp was originally identified in a porcine ETEC but is also associated with ETEC that cause disease in humans^3,4^. ETEC adhere to the small intestine through fimbrial, fibrillar or non-fimbrial outer membrane structures called colonization factors (CFs) or coli surface antigens (CS), which are composed of polymers of major subunits, and in most cases a tip protein^5^. In total, 22 antigenically distinct CFs have been identified and characterized in human ETEC^6^ and are classified on the basis of genetic and antigenic relationships (Table S1). Three major groups of CFs have been identified, i.e. CFA/I-like, CS5-like and Class 1b, which cover most known ETEC CFs. Common combinations of CFs and toxins found in human ETEC isolates include CS1 + CS3(±CS21) LT + STh, CS2 + CS3(±CS21) LT + STh, CS5 + CS6 LT + STh, CS6 STp, CFA/I(±CS21) STh and CS7 LT. These combinations are expressed by stable ETEC lineages that have both a temporal and geographic distribution^2,6–8^. The most prevalent CFs are CFA/I, CS1-CS6 and CS21. In specific geographic areas other CFs, e.g. CS7, CS14 and CS17 are prevalent^9^. However, more than 30% of all clinical ETEC isolates lack a known CF^8,10,11^. Hence, intense efforts have been made to identify novel CFs that are frequently expressed in ETEC^8,12,13^. In this project, we have searched for novel CFs in a large whole genome sequencing (WGS) database of ETEC isolates lacking known CFs, designated “CF negative” isolates. By this approach, we have identified a thermo-regulated novel fimbrial CF that mediates binding to human intestinal enterocytes among a number of LT + STp positive ETEC isolates collected from children with diarrhea.

## Results

### Identification of a putative CF gene cluster comprising seven genes

**Figure 1:**
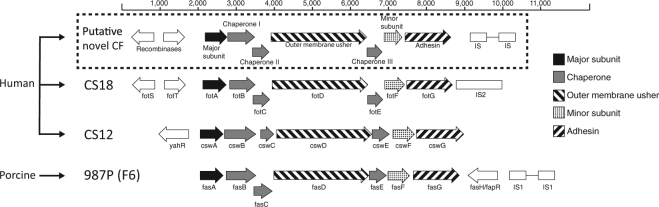
Comparison of the gene clusters of putative novel CF and related CFs. The structure of the gene cluster is based on the extracted nucleotide sequence of strain E873 harboring the putative novel CF. This gene cluster was compared to the known operon structures of related CFs, i.e. CS12, CS18 and 987P (F6).

In this study, we used a database of 94 whole genome sequenced ETEC isolates lacking a known CF (“CF negative”) to search for novel CFs. By mapping reads to a nucleotide reference database of all published known ETEC CFs (Table S1) we could identify isolates with genes or gene clusters that were similar in nucleotide sequence to known CFs. In total, we identified four “CF negative” ETEC isolates (E873, E1101, E1533 and E1586) that harbored seven genes that shared sequence homology and operon structure with the known human ETEC CFs CS12, CS18, CS20 and the porcine ETEC CF 987P (F6) (Fig. 1). In addition, the same gene cluster was identified in two ETEC isolates (E361 and E881) also harboring the genes for CS13. These CS13 positive isolates were not detected in the initial CF screen using PCR, ELISA or dotblot as there were no current CS13 specific primers or antibodies at the time. The six isolates (E361, E873, E881, E1101, E1533 and E1586) were collected from children with diarrhea in various locations worldwide between 1989 and 2003^7^ and found across the ETEC phylogeny^7^ (Table 1). The mature major subunit was predicted to have a molecular weight of 18.5 kD, similar to CS18 (18.5 kD) and 987P (17.2 kD). Two tyrosine recombinase genes were identified upstream of the putative major subunit gene.

**Table 1:** List of CS30 positive isolates identified amongst LT + STp expressing isolates.

Isolate^a^	Phylo-group	MLST	O antigen^b^	Incompatibility group^c^	Toxin Profile	Origin (Country)	Year of isolation	Subject	Age of subject	D/AS^d^
E873*	B1	940	O64	FII, FIIY, FrepB, P	LT + STp	Guatemala	2003	Indigenous	Child <5 y	D
E1101*	A	167	OC14-like^e^	FII, FIIS, FIIY, FrepB, X1	LT + STp	Egypt	2000	Indigenous	Child <5 y	AS
E1533*	A	1494	O9-like	FII, FII-s, FrepB, Y	LT + STp	Argentina	1989	Indigenous	Child <5 y	D
E1586*	A	n.d.	O9	FII, FrepB	LT + STp	Argentina	1989	Indigenous	Child <5 y	D
E361*^,■^	B1	940	O64	FII, FIIY, FrepB	LT + STp	Mexico	1998	Traveler	Adult	D/AS^f^
E881*^,■^	B1	940	O64	FII, FIIY, FrepB, P	LT + STp	Guatemala	2003	Indigenous	Child <5 y	D
E946	n.d.	n.d.	n.d.	n.d.	LT + STp	Egypt	1997	Indigenous	Child <5 y	D/AS
E1547	n.d.	n.d.	n.d.	n.d.	LT + STp	Argentina	1989	Indigenous	Child <5 y	D/AS
E2548	n.d.	n.d.	n.d.	n.d.	LT + STp	Pakistan	2009	Indigenous	Child <5 y	D/AS
E2567	n.d.	n.d.	n.d.	n.d.	LT + STp	Kenya	2009	Indigenous	Child <5 y	D/AS
E2568	n.d.	n.d.	n.d.	n.d.	LT + STp	Kenya	2009	Indigenous	Child <5 y	D/AS
E2573	n.d.	n.d.	n.d.	n.d.	LT + STp	Kenya	2009	Indigenous	Child <5 y	D/AS
E2586	n.d.	n.d.	n.d.	n.d.	LT + STp	Pakistan	2009	Indigenous	Child <5 y	D/AS
E2591	n.d.	n.d.	n.d.	n.d.	LT + STp	Pakistan	2009	Indigenous	Child <5 y	D/AS
E2612	n.d.	n.d.	n.d.	n.d.	LT + STp	Bangladesh	2009	Indigenous	Child <5 y	D/AS

^*^Whole genome sequenced isolates.

^■^Isolates also harboring CS13.

^a^Gothenburg University designation.

^b^Determined using *in silico* PCR^42^.

^c^Determined using *in silico* PCR^7^.

^d^D/AS: Diarrhea/Asymptomatic.

^e^–like: diversified O antigen.

^f^D/AS: Either diarrhea or asymptomatic.

n.d. = not determined.

The usher and the chaperone genes of fimbrial operons are known to be conserved and are used to classify fimbriae^14^. Chaperones and ushers containing the protein domains PF00577 (usher), PF02753 (chaperone) and PF02753 (chaperone) are all members of the alternate and classical usher/chaperone families^14^. These domains were identified in the usher and chaperones of the isolates harboring the novel CF, however, two candidate chaperones (I and II) contained PF02753 and one (III) contained both PF02753 and PF02753. Furthermore, the conserved protein domain PF00419 found in related ETEC CFs and Type I fimbriae was present in the major and minor subunits of the putative novel CF^14^ (Table 2).

**Table 2:** Coding DNA sequences predicted within the *csm* gene cluster and similarity to known proteins in databases.

Gene	Length (nt/aa)	Predicted protein domain (Family)	Similar protein/Organism	Function	Identity (aa)^b^	E value
*csmS*	603/200	Integrase-like catalytic domain (IPR013762)	FotS (CS18)/ETEC	Regulatory, site-specific recombinase	99%	4e-143
*csmT*	576/191	Integrase-like catalytic domain (IPR013762)	FotT (CS18)/ETEC	Regulatory, site-specific recombinase	99%	2e-137
*csmA*	606/201	Fimbrial subunit (PF00419)	CsnA (CS20)/ETEC	Fimbrial major subunit	74%	8e-97
*csmB*	708/235	PapD^a^ N terminal (PF00345), PapD C-terminal (PF02753)	FotB (CS18)/ETEC	Fimbrial chaperone	68%	2e-110
*csmC*	432/143	PapD N-terminal (PF00345)	FotC (CS18)/ETEC	Fimbrial chaperone	64%	3e-46
*csmD*	2508/835	Fimbrial usher (PF00577)	FotD (CS18)/ETEC	Fimbrial usher	77%	0.0
*csmE*	411/136	PapD N terminal (PF00345)	FotE (CS18)/ETEC	Fimbrial chaperone	90	2e-77
*csmF*	537/178	Fimbrial subunit (PF00419)	FotF (CS18)/ETEC	Fimbrial subunit	94%	3e-103
*csmG*	1191/396	Fimbrial subunit (PF00419)	FotG (CS18)/ETEC	Fimbrial adhesin	97%	0.0

^a^PapD encodes the chaperone of the Pap pili.

^b^Identity: number of matching amino acids/total number of amino acids. Includes signal peptides.

### The novel CF is closely related to the human CFs, CS18 and CS20 as well as the porcine CF 987P

**Figure 2:**
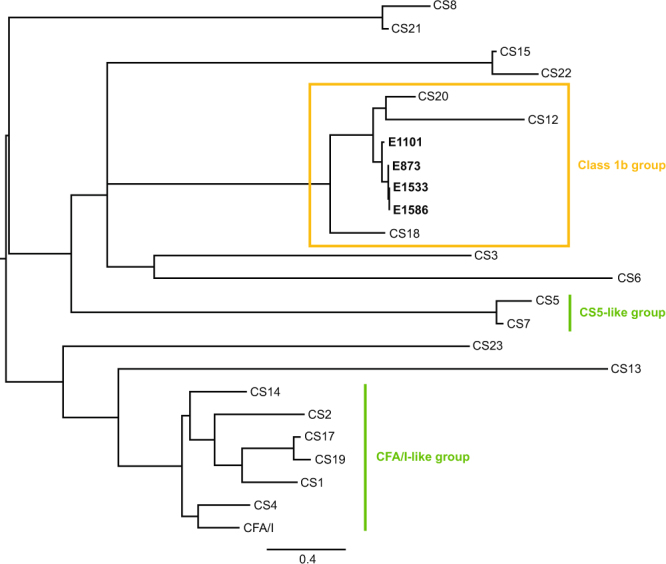
Phylogenetic tree derived from the alignment of the structural subunits of the putative major subunit and 22 defined human CF major subunits. The sequence encoding the putative major subunit in the four identified isolates (E873, E1101, E1533 and E1586) was extracted and translated. The putative major subunits cluster closely together with major subunits of defined CFs part of the Class 1b groups, indicated in orange. The protein sequence encoding the major subunit of 22 defined human ETEC CFs from NCBI Genbank was included. The two major CF groups (CFA/I-like and CS5-like groups) are indicated in green. For accession numbers see Table S1. The signal peptide was removed from all protein sequences.

A phylogenetic analysis based on the amino acid sequence encoding the putative major subunit of the novel CF including the major subunits of all known and defined ETEC CFs showed that the major subunit of the four isolates harboring the novel CF alone formed a distinct cluster together with the related CFs of Class 1b group (Fig. 2). The identified major subunit shared the highest level of amino acid sequence similarity with CsnA of CS20 (77.5–76.0%) and FotA of CS18 (68.0–64.9%), whereas the similarity with the major subunit of FasA of the porcine CF 987P was ~58% (Fig. S1). The signal peptide of the major subunit of the novel CF is highly similar to CS18 (~88%), whereas it is only 43% and 48% similar to the signal peptides of CS20 and 987P (F6), respectively. The GC content of the gene cluster of the novel CF was estimated to be 37.5% compared to the overall GC content of the chromosome being 48.4%. Thus, the sequence analyses suggest that the identified gene cluster encodes a novel CF. We have designated the novel CF as CS30 which is encoded by the genes *csmA-G*, with two recombinases named *csmS* and *csmT*.

### CS30 is a thermo-regulated CF

**Figure 3:**
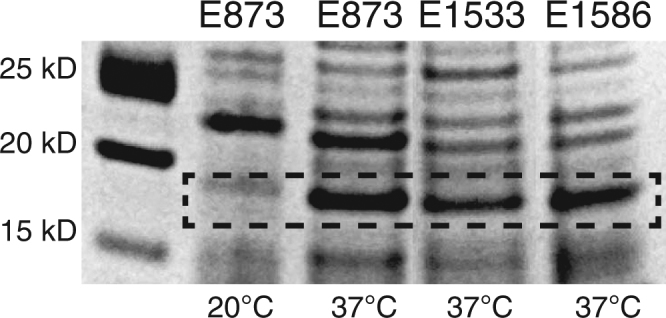
SDS-PAGE analysis of bacterial heat extracts of CS30 positive isolates. Heat extracts from isolates E873, E1533 and E1586 cultured at 20°C (only E873 shown) or at 37°C were run in an SDS-PAGE. Protein staining revealed a strong band of ~18 kD for all isolates cultured at 37°C, which was absent after culture at 20°C.

Since ETEC CFs have been shown to be thermo-regulated^13,15–17^ we analyzed protein heat extracts of the CS30-only positive ETEC isolates cultured at 20°C or 37°C by SDS-PAGE. After Commassie blue staining a clear ~18 kD polypeptide was visible for the 37°C-grown bacterial preparations, which was absent for the 20°C-grown extracts (Fig. 3). Mass spectrometry (MS) analyses of the ~18 kD polypeptide region confirmed the presence of CS30-related peptides. In addition, quantitative MS (QMS) was performed and showed that genes encoding the major and minor subunit of the CS30 operon were up-regulated 2 to 3-fold in isolates cultured at 37°C compared to at 20°C (Table S2). These results were also verified at the transcriptional level by quantitative real-time PCR (qRT-PCR) where expression of *csmA* peaked at mid log-phase at OD (OD600 ~0.9) at 37 °C (Fig. S2). Expression of *csmA* at in mid log-phase was 34-fold higher when cultured at 37 °C compared to at 20 °C (data not shown).

### CS30 mediates adhesion to Caco-2 cells and has a fimbrial structure

**Figure 4:**
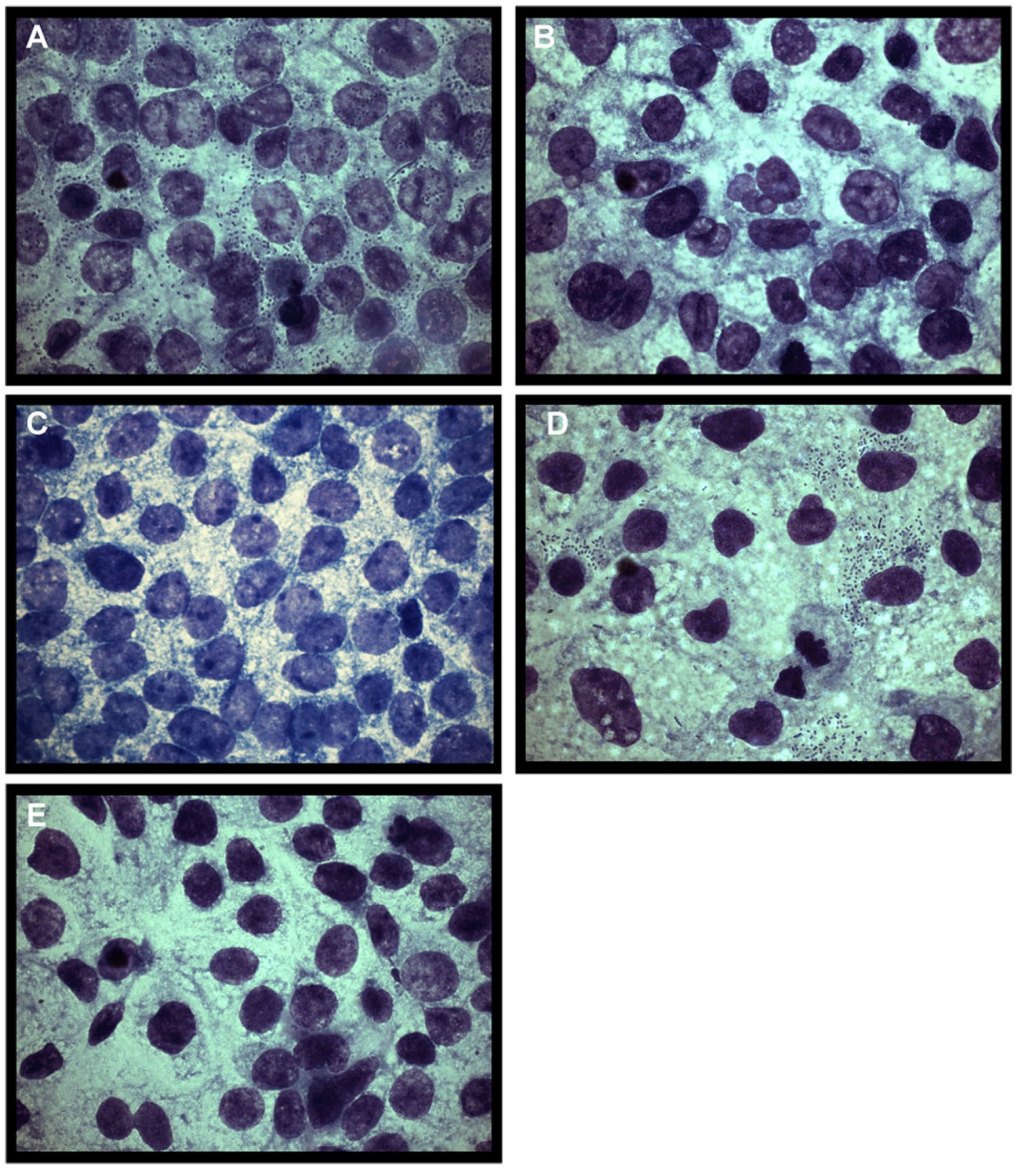
Adherence of CS30-positive ETEC isolates to Caco-2 cells. (**A**) Adherence was clearly visible when the wild type strain E873 was cultured at 37°C. (**B**) In contrast, the wild type strain E873 cultured at 20°C did not adhere to the cells. (**C**) A mutant E873 Δ*csmA* with a disrupted *csmA* encoding the major subunit did not bind significantly. (**D**) Complementation of mutant E873 Δ*csmA* with a plasmid containing the *csmA* (pMT-csmA) resulted in bound bacteria after culture at 37°C in the presence of IPTG, (**E**) and without IPTG no binding occurred. Strain E873 was used as a representative strain for all four identified CS30 positive strains (E873, E1101, E1533 and E1586).

**Figure 5:**
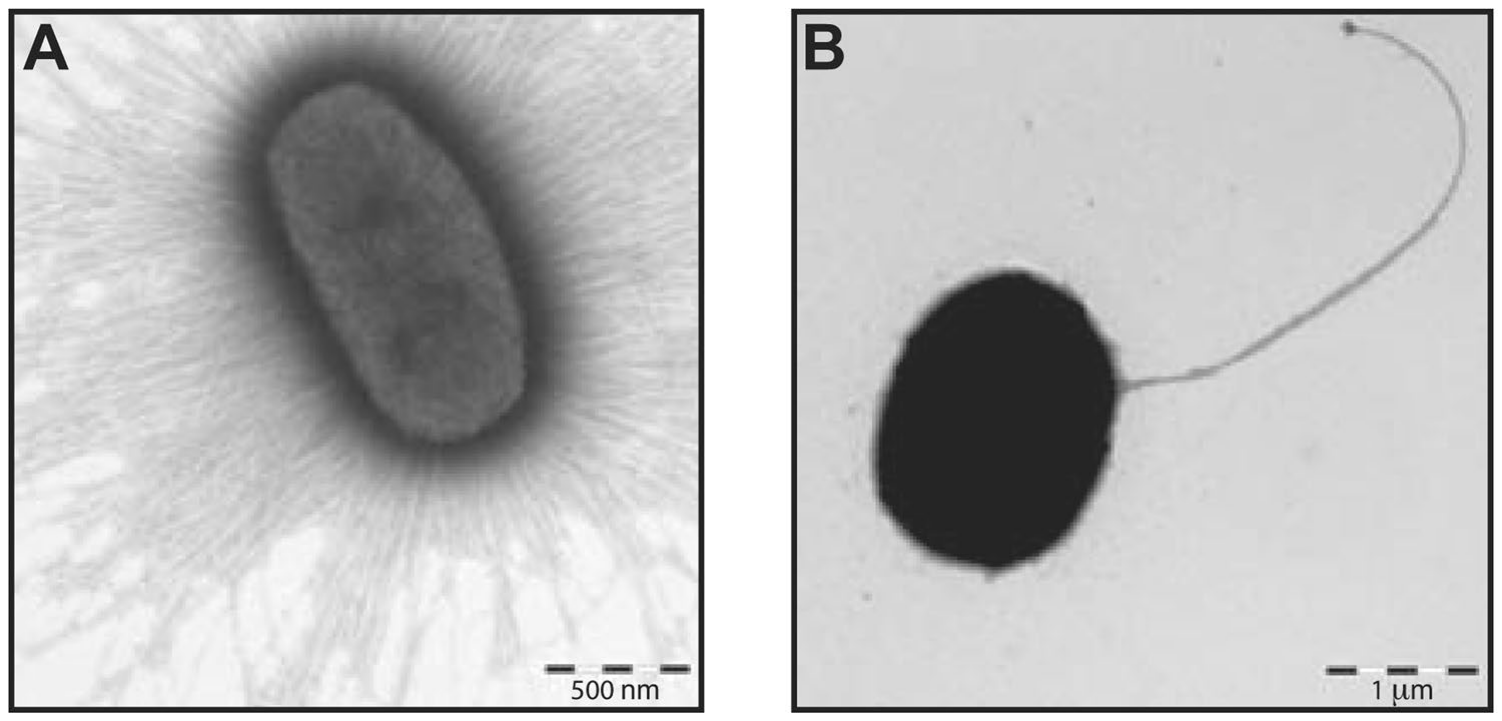
Transmission electron microscopy (TEM) analysis of E873 and E873 Δ*csmA*. Negative stain of (**A**). the wild type isolate E873 harboring CS30 shows heavily fimbriated bacteria (CS30) and of (**B)**. the E873 Δ*csmA* mutant lacking visible fimbriae.

Since ETEC CFs promote adherence to the brush border of small intestine epithelial cells we studied whether CS30 could promote binding of these CS30 expressing ETEC to the human enterocyte-like Caco-2 cell line. The four isolates expressing CS30 alone were found to adhere to Caco-2 cells when cultured at 37°C whereas corresponding preparations cultured at 20°C did not adhere (Fig. 4A and B, respectively, and Table S3). To confirm that the adhesion was due to the expression of CS30, a mutant with the major subunit (*csmA*) disrupted, E873 ΔcsmA, was generated and showed no adherence capacity to Caco-2 cells (Fig. 4C and Table S3). The adhesion to Caco-2 cells was partially recovered by introducing a plasmid into the mutant where the *csmA* gene was under the control of a tac promoter. The recovered strain (E873 ΔcsmA pMT-csmA) was shown to adhere to Caco-2 cells when cultured in the presence of IPTG and no adhesion was observed in the absence of IPTG (Fig. 4D and E, respectively, and Table S3) (for details see Material and Method section and Fig. S3 and Table S4). Transmission electron microscopy imaging showed that CS30 positive bacteria were associated with rigid fimbriae of ~7 nm in diameter and that the mutant E873 ΔcsmA derivative lacked visible fimbriae (Fig. 5).

### Genes encoding CS30 are located on a plasmid together with the enterotoxin genes

**Figure 6:**
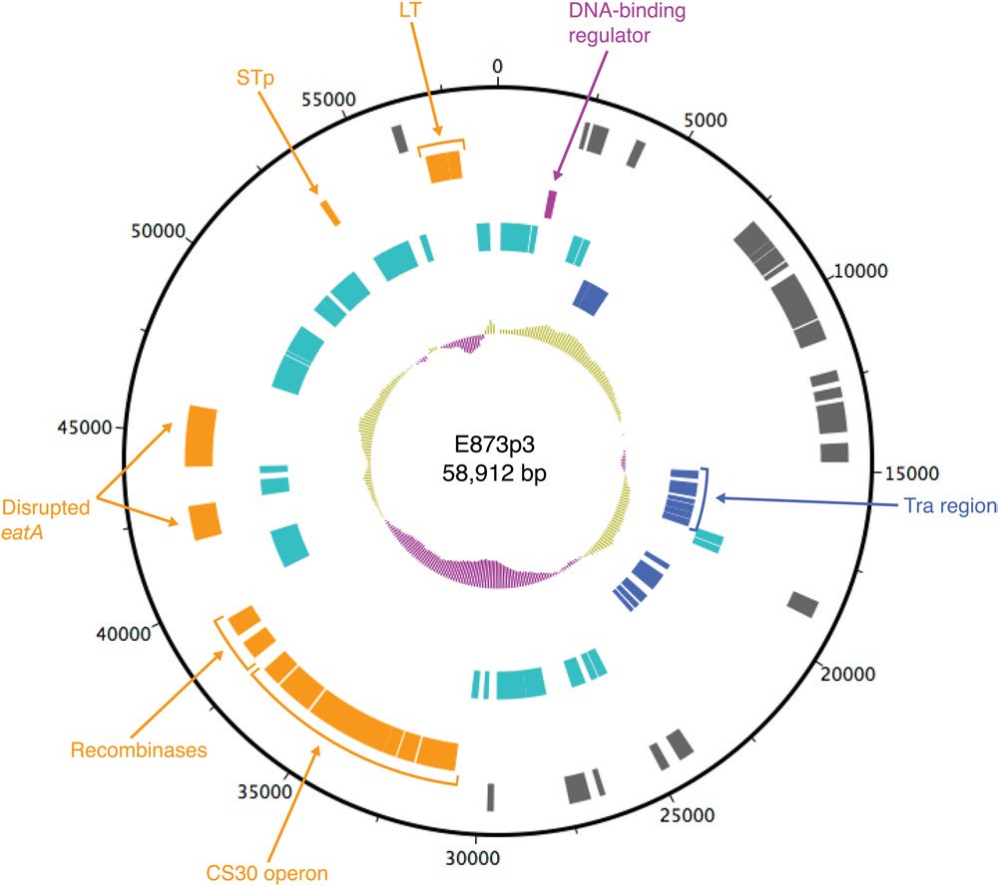
Graphical map of plasmid E873p3 harboring the CS30 gene cluster. The innermost circle represents G+C content (%) with greater (light green) to less (purple) than average (0.46). The tracks from the inside represent: (1) replication and plasmid maintenance genes; (2) insertion sequences (IS) and transposon related genes; (3) regulatory genes; (4) virulence genes; (5) other coding DNA sequences (CDS). The genes mentioned in the text are indicated with arrows with the same color as their CDS.

Single-Molecule Real Time (SMRT) sequencing (Pacific Bioscience) of DNA prepared from isolate E873 was used to show that the genes encoding CS30, together with the genes encoding, LT and STp, were located on a F-like plasmid (E873p3) carrying the FII replicon (Fig. 6 and Table 1). In total, 78 coding DNA sequences (CDS) were predicted in E873p3. In addition to the CF and toxin encoding genes plasmid specific genes included five transfer (*tra*) associated genes (*traM*, *traY*, *traA*, *traL* and *traE*), replication (*rep*) genes and plasmid stability (*sta*) genes and a gene encoding a putative regulator, i.e. a DNA-binding regulator. Furthermore, the plasmid contained a disrupted *eatA* gene encoding a serine protease that degrades mucin aiding the toxins to come in close vicinity to the intestinal cells^18,19^. The inverted repeats for the *oriT* of F plasmids has been described previously^20^ and was identified in E873p3 upstream of *traM*. Furthermore, 28 CDSs were predicted as insertion sequence (IS) elements and transposon-related genes.

### CS30 is common among “CF negative” ETEC isolates

Primers specific for the gene encoding the major subunit, *csmA*, in CS30 were designed (Table S4) to screen 58 additional “CF negative” LT + STp isolates present in the Gothenburg ETEC strain collection^7^ for CS30. In total, 15.5% (n = 9) ETEC isolates were CS30 positive (Table 2).

## Discussion

ETEC is a pathogen with a highly variable CF repertoire and adherence is regarded a crucial step in ETEC pathogenesis. Many clinical ETEC isolates express one or more CFs but other ETEC isolates collected from patients suffering from diarrhea lack any of the known CFs. Several attempts have been made to identify novel CFs, and/or surface structures with the goal to include them in an ETEC vaccine to broaden possible protective coverage^12,13^.

We have, by a reverse genetics approach, identified a novel functional CF, designated CS30, by searching for a potential set of genes from WGS data and by performing key functional analyses. By this approach we could identify four LT + STp isolates, previously classified as “CF negative”, harboring the genes encoding CS30 and two additional isolates also harboring genes for CS30 and CS13. In addition to the six ETEC isolates found to harbor the CS30 gene cluster (w/ and w/o CS13), we identified seven additional isolates harboring CS30 only by PCR analysis among 58 LT + STp “CF negative” ETEC isolates (Table 2). Thus, as many as 8.6% (n = 13) of “CF negative” (n = 152) and 19.4% (n = 13) of “CF negative” LT + STp (n = 67) isolates were positive for CS30. These strains have been isolated in different continents and have been circulating for some time.

The novel CF CS30 was shown to be related to the porcine CF 987P, sharing more than half of the amino acid sequence of the major subunit of FasA (987P). CF 987P have previously been shown to be related to the human CFs CS12, CS18 and CS20 part of the Class 1b^6,12^. Furthermore, the CF CS30 belong to the γ_2_-fimbriae family of chaperone/usher-assembled fimbriae^14^ and carries specific protein domains together with the other CFs in Class 1b family. Other ETEC CFs, such as CS13 and CS23, are also known to be related to a porcine ETEC CF, i.e. K88 (F4)^13^. In addition, the identified CS30 isolates were positive for both LT and STp. STp may be produced by ETEC infecting both humans and piglets^2^. These findings suggest that CS30 have evolved from a common ancestor related to 987P, and may have changed its host specificity over time. The potentially altered host specificity may be due to mutations in the major subunit and/or the adhesin known to be responsible for the binding to intestinal receptors^21^. Other human ETEC CFs part of the Class 1b group and related to CS30 also produce STp, supporting the hypothesis that porcine related CFs carried by plasmids can be transferred by horizontal transfer.

By means of transmission electron microscopy we could show that CS30 positive isolates were heavily fimbriated, expressing rigid fimbrial structures of ~7 nm in diameter on the bacterial surface, similar to CS18^15^.

Generation of a mutant with a disrupted major subunit, *csmA*, had no visible fimbriae and did not adhere to the Caco-2 cells indicating that the assembly of the CS30 fimbriae is dependent on a functional major subunit. The lack of adhesion seen in the mutant could be partially recovered by inserting a plasmid (pMT-csmA) containing the gene, *csmA*, encoding the major subunit under the control of a tac promoter inducible by IPTG (Table S3).

Most ETEC CFs are thermo-regulated^6,13,15,16^, i.e. culturing ETEC isolates at 37°C results in CF expression, whereas no or little expression occur after culturing at 20°C. Similar temperature-dependent regulation was observed for expression of CS30, as an ~18 kD band identified as the major subunit of CS30, was observed in SDS-PAGE for 37°C-grown bacterial preparations but not in the 20°C-grown bacterial preparations. The same cultures were used for TEM imaging and Caco-2 cell adhesion assays. The results were conclusive, visible fimbriae and adherence to Caco-2 cells were seen in bacteria cultured at 37 °C but not at 20 °C. qRT-PCR analysis showed that the expression levels of *csmA* peaked at mid log growth phase, after 3 hours (OD ~0.9) (Fig. S2), whereas no expression was observed when examining bacteria at the same OD after cultivation at 20°C.

Two site-specific recombinases were identified upstream of the gene encoding the major subunit *csmA*. Such recombinases are known to be involved in phenotype variation that may be regulated by various factors, e.g. by temperature shown in CS18, P fimbriae and Type I fimbriae^22–27^. Hence, CS30 may be regulated in a similar manner.

SMRT sequencing allows generation of long reads that can be used to circularize both the chromosome and potential plasmids^28^. One of the CS30 isolates, E873, was SMRT sequenced. The plasmid (E873p3) harboring the genes for CS30 and both toxins, LT and STp, was predicted to be an F-like plasmid, which are known to carry virulence genes^29^. The CS30 locus had almost 10% lower GC content compared to the rest of the plasmid (46.4%) and was flanked by a transposon-related gene and insertion elements indicating that the genes encoding CS30 initially were introduced through horizontal transfer.

A putative novel DNA-binding protein was identified in the same plasmid but shared no sequence similarity with other known regulators of ETEC CFs, e.g. Rns and CsvR^30,31^. The serine protease, EatA, a putative virulence factor and potential vaccine target in ETEC^18,32^ was also present in E873p3. However, the gene was disrupted by an insertion element, indicating a non-functional gene. EatA is a serine protease able to degrade mucins present in the small intestine^18^. The plasmid also harbored five transfer-associated genes and a predicted *oriT* site was identified indicating that the plasmid most likely is mobilizable with the help from the transfer system from E873p1, which harbors a complete *tra* locus (data not shown).

In conclusion, we have identified a comparatively common novel CF, CS30, amongst ETEC strains with no previously described CFs. CS30 promotes binding to intestinal epithelial cells in a temperature-dependent manner. CS30 was shown to be related to the porcine CF 987P and they have most likely evolved from a common ancestor. We propose that CS30 should be included in the screenings of clinical ETEC isolates for determining its prevalence in future epidemiological studies.

## Materials and Methods

### Bacterial strains

Ninety-four LT, ST or LT + ST positive ETEC isolates lacking a known CF, “CF negative” isolates, confirmed by both PCR and ELISA against CFA/I, CS1-CS8, CS14, CS17-CS21 were whole genome sequenced as part of a larger ETEC strain collection; the isolates have been described in a previous study^7^. The isolates were further confirmed as “CF negative” by comparative genomics after whole genome sequencing. The strains had mainly been isolated from children below 5 years of age and adults in ETEC-endemic areas as well as from travelers with diarrhea. Isolates were collected in Argentina, Bangladesh, Bolivia, Egypt, Guatemala, Indonesia, Japan, Mexico and Tunisia between 1980 and 2011.

### DNA extraction and whole genome sequencing

All isolates were grown on horse blood agar plates overnight at 37°C to detect potential contamination. Only pure ETEC cultures were used for DNA extraction. For Illumina whole genome sequencing, DNA extraction was performed with the Genomic DNA kit (Promega) according to the manufacturer’s instructions. For Single-Molecule Real Time (SMRT) sequencing (Pacific Bioscience) long intact strands of DNA needs to be obtained. The genomic DNA extraction was performed as follows. Isolate E873 was cultured in CFA broth overnight at 37°C followed by cell suspension in TE buffer (10 mM Tris and 1 mM EDTA pH 8.0) with 25% sucrose (Sigma) followed by lysis using 10 mg/ml lysozyme (in 0.25 Tris pH 8.0) (Roche). Cell membranes were digested with Proteinase K (Roche) and Sarkosyl NL-30 (Sigma) in the presence of EDTA. RNase A (Roche) was added to remove RNA molecules. A phenol-chloroform extraction was performed using a mixture of Phenol:Chloroform:Isoamyl Alcohol (25:24:1) (Sigma) in phase lock tubes (5prime). To precipitate the DNA 2.5 volumes 99% ethanol and 0.1 volume 3 M NaAc pH 5.2 was used followed by re-hydration in 10 mM Tris pH 8.0. DNA concentration of samples used for both Illumina and SMRT sequencing was measured using NanoDrop spectrophotometer (NanoDrop). On average 1 μg of DNA was used for Illumina sequencing and 10 μg for PacBio sequencing. A detailed description of the Illumina sequenced isolates has previously been published^7^. Library preparation for SMRT sequencing was prepared according to the manufacturers’ (Pacific Biosciences) protocol. The DNA was stored in E buffer and sequenced at the Wellcome Trust Sanger Institute. Isolate E873 was sequenced with a single SMRTcell using the P6-C4 chemistry, to a target coverage of 40–60X using the PacBio RSII sequencer.

### De novo sequence assembly and circularization

De novo assembly of the Illumina sequenced isolates have previously been described^7^. The resulting raw sequencing data from SMRT sequencing was manually de novo assembled using the PacBio SMRT analysis pipeline (https://github.com/PacificBiosciences/SMRT-Analysis) (version 2.3) utilizing the Hierarchical Genome Assembly Process (HGAP)^33^. The unfinished assembly produced a single, non-circular, chromosome plus some small contigs, some of which were plasmids or unresolved assembly variants. Using Circlator^34^ (version 1.1.0), small self-contained contigs in the unfinished assembly were identified and removed, with the remaining contigs circularized. Quiver^33^ was then used to correct errors in the circularized region by mapping corrected reads back to the circularized assembly. The final assembly was annotated using Prokka^35^ (version 1.5). Plasmid E873p3 was further annotated to include the genes encoding CS30 as well as enterotoxins LT and STp.

### Culture conditions and toxin profiles of ETEC isolates

#### Culture conditions

All isolates were grown on CFA agar plates containing 0.15% crude bile overnight at 37°C. In total, 10^7^ bacteria/ml were added to CFA broth containing 0.15% crude bile and cultured for three hours (37°C) or until OD ~0.9–1.0 was reached (20°C), which is equivalent to a three-hour culture at 37°C.

#### Toxin profile

The presence of genes encoding the toxins (LT, STh and STp) was confirmed by genomic analyses and PCR and toxin production was confirmed by LT GM1 and ST Inhibition GM1-ELISA as previously described^36,37^.

### Cell cultures

Intestinal Caco-2 cells, clone C2BBbe1 clone (ATCC), derived from human colonic carcinoma, were cultured in DMEM/F-12 (Life Technologies Invitrogen) supplemented with 10% fetal bovine serum (Fisher Scientific) and 10 µg/ml Apo-transferrin solution (Sigma Aldrich) at 37 °C in 5% CO_2_. To propagate cells, they were seeded in 25 cm^2^ flasks and grown to 70% confluence. Cells were detached with 0.25% Trypsin-EDTA (Sigma-Aldrich) and split appropriately. Upon cell adhesion assays, 4 × 10^4^ cells were seeded into 8 well-chamber glass-slides (NUNC) and cultured past confluence and for an additional 15 days for proper differentiation. Culture medium was changed every two days.

### Caco-2 cell adhesion assay

The ETEC isolates were cultured as described above. Cultured bacteria were washed once in phosphate buffered saline solution (PBS) and the number of bacteria was set by OD_600_ measurement. In total, 10^9^ bacteria/ml in DMEM/F-12 + 0.5% D-mannose (Sigma Aldrich), to inhibit binding of mannose-sensitive Type I fimbriae, were added to the wells, which contained a confluent layer of washed Caco-2 cells, and incubated for three hours at 37°C. The infected cells were gently washed with DMEM/F-12 three times to remove non-adherent bacteria. The Caco-2 cells with adhering bacteria were then fixated with 70% ethanol for ten minutes and subsequently stained with 10% Giemsa solution (VWR) for 15 minutes. The stained cells were washed and allowed to dry overnight followed by analysis by light microscopy (Zeiss Axioskop 2 mot plus). The adhesion assays for each strain was repeated at least three times and each strain tested in the assay was microscopically examined blindly to avoid potential observer bias. The adhesion index was determined as the percentage of Caco-2 cells with at least one adhering bacterium in five randomly chosen microscopic fields. The strain was regarded positive if more than 10% of the Caco-2 cells had adhering bacteria. The cut-off value was determined by the adhesion index plus two standard deviations from an in house CFA/I negative reference strain^38^ used in the Caco-2 cell adhesion assay.

### Expression of CS30

#### Growth conditions

A growth curve of E873 expressing CS30 was conducted to evaluate the time-point for the highest expression of CS30. The bacteria were cultured as described above and samples were collected every hour during seven hours.

#### RNA extraction

Total RNA was prepared from a volume corresponding to 10^9^ bacteria, treated with RNAProtect after collection and stored at −70°C until use, with the RNeasy Mini Kit (Qiagen), removing contaminating DNA on-column by using the RNase-Free DNase Set (Qiagen). The integrity of the RNA and absence of contaminating DNA was checked by agarose gel electrophoresis and the RNA concentration was measured at 260/280 nm using NanoDrop spectrophotometer. All RNA samples were stored at −70°C.

#### cDNA synthesis and Quantitative Real-Time PCR (qRT-PCR)

cDNA was synthesized from up to 600 ng total RNA using QuantiTect Reverse Transcription Kit (Qiagen), including an additional DNase treatment step. Controls without reverse transcriptase (−RT) was prepared simultaneously with the synthesized cDNA from the same amount of RNA. The qRT-PCR was performed using primers specific for the gene encoding the major subunit (*csmA*) of CS30 (Table S4). The qRT-PCR program was run in 20 μl reactions under standard conditions for the ABI 7500 (Applied Biosystems), with 30 ng cDNA, 8 pmol of each primer and 10 μl Power SYBR®Green PCR Master Mix (Applied Biosystem). The levels of transcripts were normalized against the sample collected after two hours of culture.

### Transmission electron microscopy

Bacteria were cultured as described above. In total, ca. 1.6 × 10^10^ washed bacteria were applied to glow-discharged Formvar/Carbon copper-coated grids and fixated with 2% glutaraldehyde (Sigma Aldrich) followed by negative stain by 0.5% phosphotungstic acid (Sigma Aldrich) pH 6.5. Imaging analyses were made on a LEO 912AB transmission electron microscope (Zeiss) equipped with a Veleta 2 × 2k CCD camera (Olympus-SiS). The iTEM software (Olympus-SiS) was used for microscope control and image capture.

### SDS-PAGE

Bacterial heat extracts were prepared: one volume “spun down bacteria” and two volumes PBS were heated at 60°C for 30 minutes followed by centrifuging at 5000 g for 10 minutes. The supernatants were applied on a NuPAGE 10% Bis-Tris Gel (Novex) according to the manufacturers manual. The gels were stained with Commassie brilliant blue (Bio-Rad) and protein bands visible in the region 12–25 kD for bacteria cultured at 37°C which was absent at 20°C were identified.

### Mass spectrometry

#### Study group

In total, eight samples were analyzed encompassing the four isolates harboring CS30 cultured at 37°C or 20°C.

#### Proteomic analysis

Cell pellet were lysed in 60 µl lysis buffer (50 mM Triethylammoinium bicarbonate (TEAB) (Fluka, Sigma Aldrich), 2% Sodium dodecyl sulfate (SDS)) followed by protein determination with Pierce™ BCA Protein Assay (Thermo Scientific). 30 µg of total protein of each sample were reduced by addition of DL-Dithiothreitol (DTT, final concentration 100 mM) followed by trypsin digested using the filter-aided sample preparation (FASP) method modified from^39^. In short, reduced samples diluted with 400 µl 8 M urea were applied on Nanosep 30 k Omega filters (Pall Life Sciences) and 8 M urea were used to repeatedly wash away the SDS. Alkylation was performed with methyl methane thiosulfonate (MMTS, final concentration 10 mM) diluted in digestion buffer (1% sodium deoxycholate (SDC), 20 mM TEAB) and the filters were repeatedly washed with digestion buffer. Trypsin (Pierce Trypsin Protease, MS Grade, Thermo Fisher Scientific) in a ratio of 1:100 relative to protein amount was added with 50 mM TEAB to a pH 8 and the samples were incubated at 37°C for three hours. Another portion of trypsin were added and incubated overnight at 37°C. The peptides were collected by centrifugation and subjected to isobaric mass tagging reagent TMT® according to the manufacturer’s instructions (Thermo Scientific). Each sample was labeled with a unique tag from a TMT 10plex isobaric mass tag labeling kit. After TMT labeling the samples were combined, concentrated to ~150 µl in a vacuum centrifuge and acidified with formic acid to pH 2 to precipitate SDC. The peptide samples were further fractionated with High pH Reversed-Phase Spin Columns (Thermo Scientific) according to the manufacturer’s protocol.

#### LC-MS/MS Analysis

The eight fractions of the 10-plexed TMT-labeled sample was reconstituted with 15 µl of 0.1% formic acid (Sigma Aldrich) in 3% acetonitrile and analyzed on an Orbitrap Fusion Tribrid mass spectrometer interfaced to an Easy nanoLC1000 (Thermo Fisher Scientific). Peptides (2 µL injection volume) were separated using an in-house constructed analytical column (300 × 0.075 mm I.D.) packed with 1.8 μm Reprosil-Pur C18-AQ particles (Dr. Maisch). Solvent A was 0.2% formic acid in water and solvent B was 0.2% formic acid in acetonitrile. The following gradient was run at 200 nL/min; 5–30% B over 75 min, 30–80% B over 5 min, with a final hold at 80% B for 10 min. Ions were injected into the mass spectrometer under a spray voltage of 1.6 kV in positive ion mode. MS scans was performed at 120,000 resolution, m/z range 380–1,200, MS/MS analysis was performed in a data-dependent multinotch mode, with top speed cycle of 3 s for the most intense doubly or multiply charged precursor ions. An inclusion list of peptides from theoretically digested proteins of interest (CsmA-G, CsmS-T, LTh and STp) were included in the method. Ions in each MS scan over threshold 5,000 were selected for fragmentation (MS2) by collision induced dissociation (CID) for identification at 30% and detection in the ion trap followed by multinotch (simultaneous) isolation of the top 10 MS2 fragment ions, with m/z 400–900, selected for fragmentation (MS3) by high energy collision dissociation (HCD) at 55% and detection in the Orbitrap at 60000 resolution, m/z range 100–500. Precursors were isolated in the quadrupole with a 1.6 m/z window and dynamic exclusion within 10 ppm during 30 seconds was used for m/z-values already selected for fragmentation.

#### Database Search for protein TMT Quantification

MS raw data files for the TMT set were merged for relative quantification and identification using Proteome Discoverer (version 1.4) (Thermo Fisher Scientific). A database search for each set was performed with the Mascot search engine (Matrix Science) using a database based on the whole genome sequences from the four tested isolates (E873, E1101, E1533 and E1586) with MS peptide tolerance of 5 ppm and MS/MS tolerance for identification of 500 millimass units (mmu). Tryptic peptides were accepted with zero missed cleavage and variable modifications of methionine oxidation, cysteine alkylation and fixed modifications of N-terminal TMT-label and lysine TMT-label were selected. The detected peptide threshold in the software was set to a significance of 99% Mascot score by searching against a reversed database and identified proteins were grouped by sharing the same sequences to minimize redundancy. For TMT quantification, the ratios of the TMT reporter ion intensities in HCD MS/MS spectra (m/z 126–131) from raw data sets were used. Ratios were derived by Proteome Discoverer using the following criteria: fragment ion tolerance as 3 mmu for the most confident centroid peak and missing values are replaced with minimum intensity. Only peptides unique for a given protein were considered for relative quantitation, excluding those common to other isoforms or proteins of the same family. The quantification was normalized using the protein median.

### Sequence analysis and phylogenetic analysis

The whole genome sequences of 94 “CF negative” isolates were compared to known CFs using Burrows-Wheeler Alignment (BWA) mapping. The results were analyzed by creating a heatmap including hits with more than 50 read coverage as a cut-off. The initial four identified isolates with a similar gene cluster structure as CS18 was further analyzed. Multiple alignments by ClustalO in SeaView (version 4.5.4) of the amino acid sequence of the major subunits from CS30, CS18, CS20 and 987P were performed. Phylogenetic group, MLST, O antigen and incompatibility profile of the six whole genome sequenced isolates was previously determined according to von Mentzer *et al*.^7^. The sequence for E873p3 was analyzed using PlasmidFinder to determine the incompatibility group and the present replicons^40^.

A maximum likelihood-based phylogenetic tree was constructed using SeaView based on a protein alignment of the major structural subunit of CS30-only isolates and 22 of the identified and characterized human CFs (Fig. 3) (for accession numbers see Table S1).

### Inactivation of the major subunit CsmA in ETEC strain E873

The inactivation was carried out using strains, primers and the plasmids as shown in Table S4, and as described previously^41^. Briefly, PCR was applied using High Fidelity taq polymerase (Roche): 95°C 5 minutes, 30 cycles of (94°C 15 seconds, 62°C 30 seconds, 72°C 25 seconds), 72°C 7 minutes, for both fragments. The two amplified fragments were then annealed in a primerless PCR with a fragment containing Kan-Cassette, by mixing equal molar amounts of each fragment (94°C 1 minutes, 60°C 1 minute, 72°C 3 minutes and 10 seconds, 8 cycles of (94°C 30 seconds, 60°C 30 seconds, 72°C 2 minutes and 10 seconds), 72°C 3 minutes). The primers For-csmA and Rev-csmA with additional taq polymerase and dNTP were then added and the PCR reaction was continued with additional 29 cycles and a final extension of 7 minutes at 72 °C. The amplified fragment was used to construct the plasmid pMT-SacB-Cm-(csmA::Kan) and for propagation in the *E*. *coli* S17-1 strain. After mating with strain E873 the homologous recombination of the plasmid with the native *csmA* was verified by PCR using the primers amplifying outside of the cloning region, (For-upstream-csmA or and Rev-Downstream-csmA) (Table S4) and sequencing. A mutant was selected and cultivated for 10 passages in presence of kanamycin, resulting in looping out of the plasmid while the kanamycin cassette was retained. A final mutant strain, i.e. E873 Δ*csmA* was selected and used for further analyses. The construction of the E873 Δ*csmA* mutant is depicted in Fig. S3. The expression of the genes encoding the CS30 fimbriae (*csmA*-*csmG*) was confirmed using regular PCR, after cDNA synthesis, using *csmA*-*csmG* specific priners. The expression of *csmA* was abolished in the mutant, E873 Δ*csmA*, by the Kanamycin cassette while the downstream genes (*csmB-G*) were expressed (Fig. S4).

### Complementation of the E873 ΔcsmA mutant

In the strain E873 Δ*csmA* in which the gene encoding the major subunit of CS30 was disrupted a plasmid (pMT-csmA) with the *csmA* gene inserted behind a tac promoter was used to recover the mutant. The *csmA* gene was expressed constitutively in presence of IPTG in the culture media. RNA was extracted and regular PCR after cDNA synthesis, using *csmA*-*csmG* specific primers, was used to check for gene expression in the complemented mutant, E873 Δ*csmA* pMT-csmA (Fig. S4). Furthermore, Caco-2 adhesion assays was used to verify the complementation of *csmA* and compared to both the wild type E873 strain and E873 Δ*csmA*.

### Accession Numbers

The sequence for the plasmid E873p3 has been submitted to the European Nucleotide Archive, accession number: LT174529.

## Electronic supplementary material

Supplementary Information
