# Publisher Correction: Identification and characterization of the novel colonization factor CS30 based on whole genome sequencing in enterotoxigenic *Escherichia coli* (ETEC)

**DOI:** 10.1038/s41598-018-23723-6

**Published:** 2018-04-12

**Authors:** Astrid von Mentzer, Joshua Tobias, Gudrun Wiklund, Stefan Nordqvist, Martin Aslett, Gordon Dougan, Åsa Sjöling, Ann-Mari Svennerholm

**Affiliations:** 1 Department of Microbiology and Immunology, Sahlgrenska Academy, University of Gothenburg, Gothenburg, Sweden; 2 Pathogen Genomics, The Wellcome Trust Sanger Institute, Hinxton, Cambridge, United Kingdom; 3 Microbial Pathogenesis Group, The Wellcome Trust Sanger Institute, Hinxton, Cambridge, United Kingdom; 4 Department of Microbiology, Tumor and Cellbiology, Karolinska Institutet, Stockholm, Sweden

Correction to: *Scientific Reports* 10.1038/s41598-017-12743-3, published online 02 October 2017

In the original version of this Article, the author Astrid von Mentzer was incorrectly indexed. This error has now been corrected.

