# Supplementary material for: Identification and characterization of the novel colonization factor CS30 based on whole genome sequencing in enterotoxigenic Escherichia coli (ETEC)
Source: Sci Rep. 2017 Oct 2;7:12514. doi: 10.1038/s41598-017-12743-3 (PMC5624918; doi:10.1038/s41598-017-12743-3)
Supplement: Supplementary file 1 — Supplementary Information [file 41598_2017_12743_MOESM1_ESM.pdf]

Supplementary Information

Identification and characterization of the novel colonization factor CS30 based on whole genome sequencing in enterotoxigenic *Escherichia coli* (ETEC)

Astrid von Mentzer[1,*], Joshua Tobias[1], Gudrun Wiklund[1], Stefan Nordqvist[1], Martin Aslett[2], Gordon Dougan[3], Åsa Sjöling[4], Ann-Mari Svennerholm[1]

[1]Department of Microbiology and Immunology, Sahlgrenska Academy, University of Gothenburg, Sweden
[2]Pathogen Genomics, The Wellcome Trust Sanger Institute, Hinxton, Cambridge United Kingdom
[3]Microbial Pathogenesis Group, The Wellcome Trust Sanger Institute, Hinxton, Cambridge United Kingdom
[4]Department of Microbiology, Tumor and Cellbiology, Karolinska Institutet, Stockholm, Sweden
[*] astrid.von.mentzer@gu.se

Supplementary Information

FIGURES

```
Putative major subunit  APADNDASKATLNFSGRVTSSLCQVKTDDLTKDISLGEVSKSALAASGKGPAQSFQVNLI   60
FasA 987P               APAENNTSQANLDFTGKVTASLCQVDTSNLSQTIDLGELSTSALKATGKGPAKSFAVNLI   60
                        ***:*::*:*.*:*:*:**:*****.*.:*:: *.***:*.*** *:*****:** ****

Putative major subunit  NCDTTTNDISYVLADANGNGNGANGASTYLVPKSGDTAAEGVGVFVETSNGTKVNIGTAQ   120
FasA 987P               NCDTTLNSIKYTIAGNNNTG----SDTKYLVPASNDTSASGVGVYIQDNNAQAVEIGTEK   116
                        ***** *.*.*.:*  * .*    . :.**** * **:*.****::: .*.  *:*** :

Putative major subunit  TLNVVSNGATALSEQVIPLRAYIGTQNGTGGTIGTNGLKAGTVDATGVLTIRANYKANTP   180
FasA 987P               TVPVVSNGGLALSDQSIPLQAYIGTTTGNPDT--NGGVTAGTVTASAVMTIRSAGTP*--   171
                        *: *****. ***:* ***:***** .*.  *  . *:.**** *:.*:***:  .

Putative major subunit  *   180
FasA 987P               -   171
```

Figure S1. Amino acid sequence comparison between the putative major subunit and the major subunit FasA (987P). The major subunits share 58.3% of the amino acid sequence. Isolate E873 was used as a representative for all isolates harboring the novel CF. Deduced amino acid sequences were aligned using ClustalO. Amino acids that are identical (*), strongly similar (:), weakly similar (.) or different ( ) are indicated.

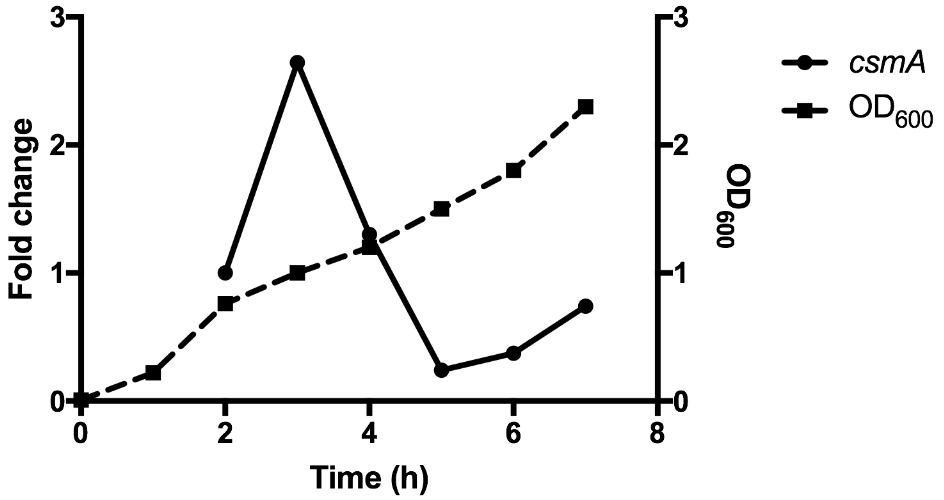

Figure S2. Expression of the *csmA* gene in E873. Dotted curve shows the growth curve (OD_{600} = 0.9) of isolate E873 cultured at 37°C and the solid line the gene expression of *csmA* measured by qRT-PCR during seven hours. The highest expression was seen after three hours. Fold change is calculated relative to the expression levels at 2 hours at 37°C. Expressions at 20°C was virtually undetectable (not shown).

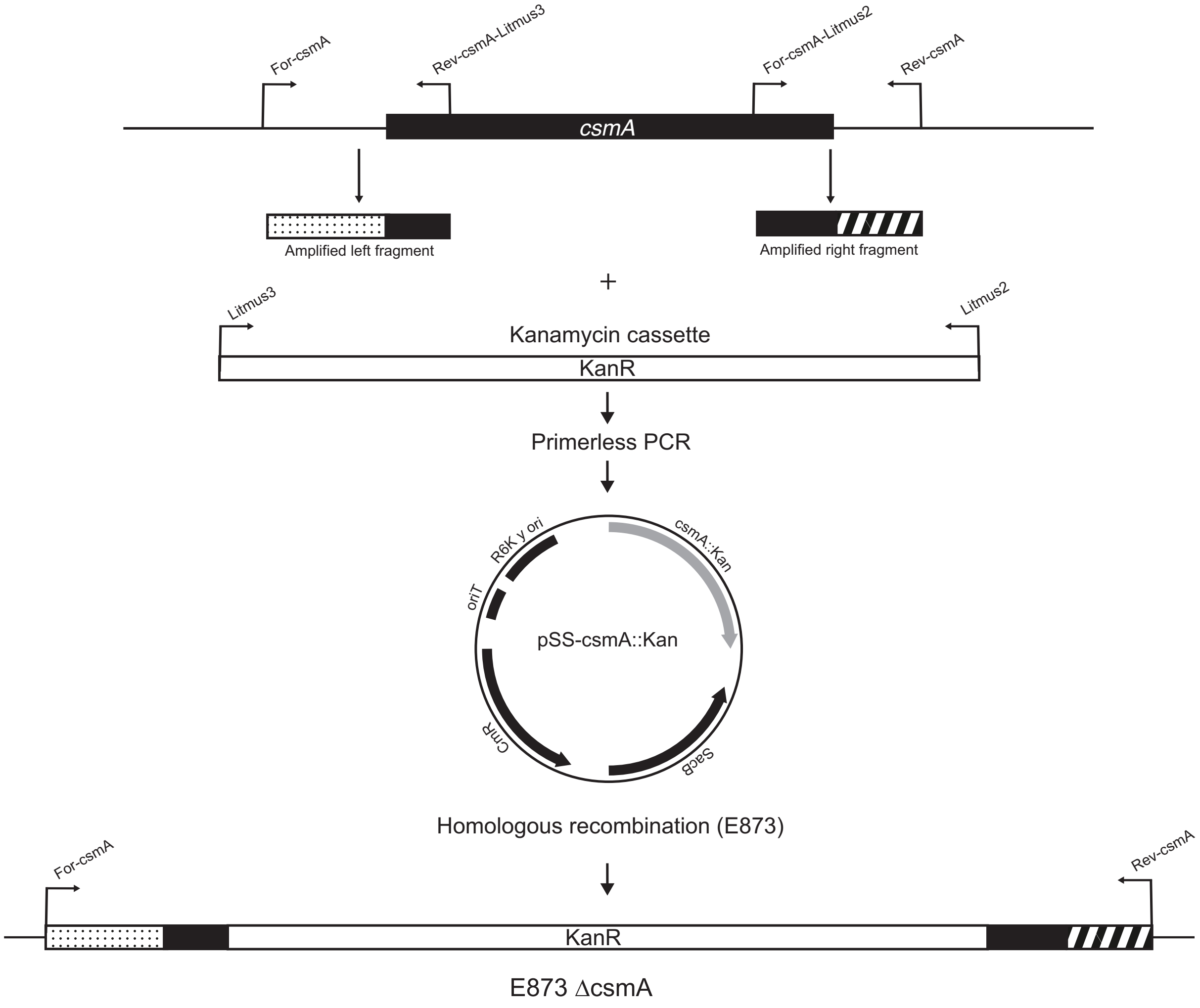

Figure S3. A schematic figure over the construction of the E873 ΔcsmA mutant. Amplification of the left and the right fragment was performed to fuse together with pMT-suicide-SacB (pSS) in a primerless PCR. Incorporation of the csmA::Kan fragment was integrated into the pSS followed by integration by homologous recombination with the plasmid carrying CS30.

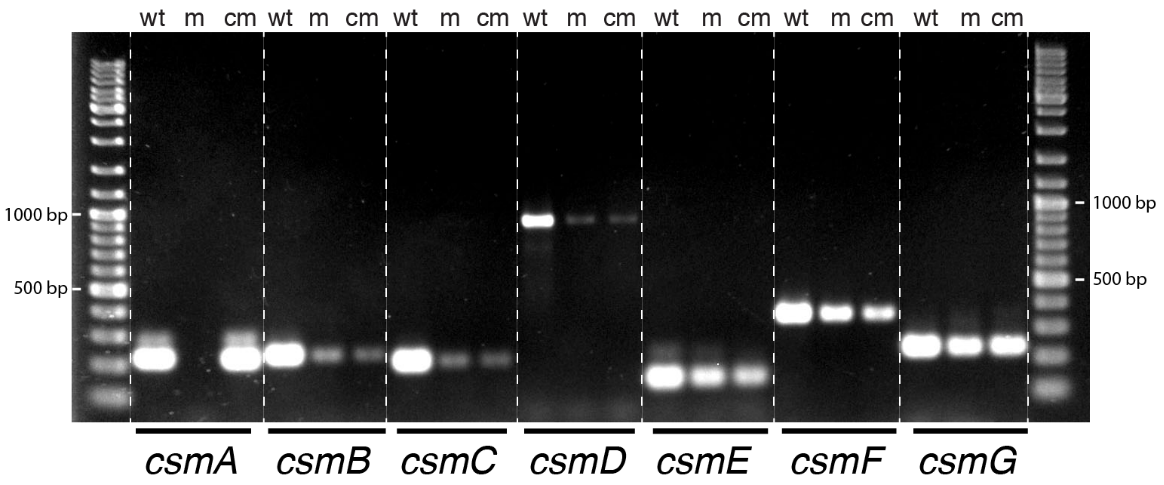

Figure S4. Gene expression of *csmA-G*. Specific primers were used to amplify fragments within the genes encoding CsmA-G by regular PCR. The major subunit (*csmA*) is not expressed in the mutant (m), E873 ΔcsmA, where a Kanamycin cassette was inserted. The wildtype strain (wt), E873, and the complemented mutant (cm), E873 ΔcsmA pMT-csmA, express all genes (*csmA*-*csmG*). PCR products: *csmA* = 218 bp; *csmB* = 230 bp; *csmC* = 208 bp; *csmD* = 879 bp; *csmE* = 142 bp; *csmF* = 348 bp; *csmG* = 232 bp.

TABLES

Table S1. Characteristics of human ETEC CFs.

CF	Morphology[a,1]		Size (kD)[b,1]	Accession number	Refs
**CFA/-like group**					
CFA/I	F	7 nm	25.0	M55661.1	[2,3]
CS1	F	7 nm	15.2	AY536429.1	[4-7]
CS2	F	7 nm	15.4	Z47800	[5,7,8]
CS4	F	6 nm	15.0	AF296132.1	[9]
CS14	F	7 nm	15.0/15.5	AY283611	[10]
CS17	F	7 nm	15.5	AY515609.1	[11]
CS19	F	7 nm	15.0	AY288101.1	[11]
PCFO71*	n.d.	n.d.	n.d.	AY513487.1	[12]
**CS5-like group**					
CS5	H	5 nm	18.6	AJ224079	[13]
CS7	H	3-6 nm	18.7	AY009095.1	[14]
**Class Ib-group[c]**					
CS12	F	7 nm	17.9	AY009096.1	[15]
CS18	F	7 nm	18.5	AF335469.1	[16]
CS20	F	7 nm	17.5	AF438155	[17]
CS26*	n.d.	n.d.	n.d.	HQ203050	[18]
CS27A*	n.d.	n.d.	n.d.	HQ203047	[18]
CS27B*	n.d.	n.d.	n.d.	HQ203048	[18]
CS28A*	n.d.	n.d.	n.d.	HQ203049	[18]
CS28B*	n.d.	n.d.	n.d.	HQ203046	[18]
**Additional**					
CS3	f	2-3 nm	15.0	FN822745.1	[5,7,19]
CS6	nF		15.1/15.9	U04844	[9,20,21]
CS8	F	7 nm	25.3	AB059751	[22]
CS21	F	7 nm	25.2	EF59570.1	[23]
CS15	nF		18.2	X65623	[24]
CS22	f	n.d.	15.0	AF145205.1	[25]
CS10	nF		16.0	n.a.	[26]
CS11	f	3 nm	n.d.	n.a.	[27]
CS13[d]	f	n.d.	24.8	X71971	[28]
CS23[d]	f/nF		16.9	JQ434477	[29]

[a] F = fimbrial; f = fibrillar; nF = non-fimbrial; H = helical.
[b] The size of the major subunit was predicted using the published amino acid sequences.
[c] All CFs in Class 1b are related to the porcine CF 987P (F6)[18].
[d] CS13 and CS23 are related to the porcine CF K88 (F4)[29].
n.d. = not determined.
n.a. = not available.
* Putative ETEC CFs

Table S2. Peptides identified by quantitative mass spectrometric (QMS) analyses in bacterial cultures grown at 37°C or 20°C.

Protein	Peptide Sequences	ETEC isolates			
		E873<br>37°C/20°C	E1101<br>37°C/20°C	E1523<br>37°C/20°C	E1586<br>37°C/20°C
CsmF	NTVLNFTENSSVK	1.6*	2.2	3.1	2.9
CsmA	VTSSLCQVK<br>SALAASGK<br>AGTVDATGVLTIR<br>ATLNFSGR	2.3	2.0	2.9	2.9

*Numbers indicate the ratio of peptides identified in the samples cultured at 37°C and 20°C.

Table S3. Adhesion of strains E873 (CS30), E873 ΔcsmA and E873 ΔcsmA pMT-csmA to Caco-2 cells

Strains	Caco-2 cells
	% cells with adherent bacteria[b]
E873 CS30 37°C[a]	92.9%±1.9
E873 CS30 20°C[a]	4.2%±2.6
E873 ΔcsmA	2.9%±0.65
E873 ΔcsmA pMT-csmA +IPTG	28.3%±12.5
E873 ΔcsmA pMT-csmA –IPTG	4.5%±2.8

[a] Strain E873 was used as a representative strain for all four identified CS30 positive strains. Similar adhesion indexes were seen for all CS30 positive strains.

[b] Percent mean ± of cells with at least one adhering bacterium.

Table S4. Strains, plasmids and primers used in the study.

Strains	Relevant characteristics	Reference
E. coli S17-1	λpir, auxotrophic to Proline	Supplied by M. Lebens
E. coli S17-1- (csmA::Kan)	λpir, auxotrophic to Proline, harboring pJT-SacB-Cm-(csmA::Kan)	This study
ETEC E873	LT, STp, CS30	icddr,b, Dhaka, Bangladesh
ETEC E873 (ΔcsmA)	LT, STp, CS30 (csmA::Kan)	This study
ETEC E873 (ΔcsmA pMT-csmA)	LT, STp, CS30 (csmA::Kan), pMT-csmA	This study
**Plasmids**		
pMT-SacB-Cm	Cm, suicide plasmid	Supplied by M. Lebens[30]
pMT-SacB-Cm-(csmA::Kan)	Cm, Kan, LT::Kan	This study
pMT-ctxA	Cm	Supplied by M. Lebens
**Primers***	**Sequence (5'-3')**	**Product (bp)**
	CS30 detection	
For-csmA	AGTCAGCTCTTGCAGCCAGT	219
Rev-csmA	CCTTGGTACCATTGCTGGTT	
For-csmB	ATCCGTGTTCTCTGTTCGGG	230
Rev-csmB	ACCATTCAAGGCTTTCGGGT	
For-csmC	GTGCAAGAGTTAGGTGTTGCTG	208
Rev-csmC	GCGCTCGGCTTCTTTTCTTT	
For-csmD	TATTCGAGAGGCTGACGGGA	879
Rev-csmD	TTATCGTTCCCCCAACTGCC	
For-csmE	ACCCAGGAAGTTTGGTTTGGT	142
Rev-csmE	TCAGGAGTGCTTTTCGGGTA	
For-csmF	AGTTAGCGAACGGGGATCAA	348
Rev-csmF	TATCTGTCGGGACGACTTGC	
For-csmG	TGCTAATGACGGCACAGGAG	232
Rev-csmG	CATGCGATAATACGCCCCCT	
	Kanamycin insertion	
For-csmA	CCACTTTCTTCCAGCAACCA	
For-csmA-Litmus3	CTGGCGTAGCTTGGCGTAATCATGGGTCACACGCCCTGAAAAGTT	
Rev-csmA	TGAGGGCTCTACCCTGAAAA	
Rev-csmA-Litmus2	CTGGCGTAATAGCGAAGAGGCCCTGCGTGCCTACATTGGTACT	
For-upstream-csmA	TGCAACGCAGTGCTTAAATC	
Rev-Downstream-csmA	CATCACCCGAACAGAGAACA	
Litmus 3	GGGCCTCTTCGCTATTACGCCAG	
Litmus 2	CCATGATTACGCCAAGCTACGCCAG	

*All primers have been designed in this study.

REFERENCES

1. Gaastra, W. & Svennerholm, A. M. Colonization factors of human enterotoxigenic *Escherichia coli* (ETEC). *Trends Microbiol* **4,** 444–452 (1996).
2. Evans, D. G., Evans, D. J., Tjoa, W. S. & DuPont, H. L. Detection and characterization of colonization factor of enterotoxigenic *Escherichia coli* isolated from adults with diarrhea. *Infect Immun* **19,** 727–736 (1978).
3. Jordi, B. J., Willshaw, G. A., van der Zeijst, B. A. & Gaastra, W. The complete nucleotide sequence of region 1 of the CFA/I fimbrial operon of human enterotoxigenic *Escherichia coli*. *DNA Seq* **2,** 257–263 (1992).
4. Marron, M. B. & Smyth, C. J. Molecular analysis of the cso operon of enterotoxigenic *Escherichia coli* reveals that CsoA is the adhesin of CS1 fimbriae and that the accessory genes are interchangeable with those of the cfa operon. *Microbiology* **141 (Pt 11),** 2849–2859 (1995).
5. Evans, D. G. & Evans, D. J. New surface-associated heat-labile colonization factor antigen (CFA/II) produced by enterotoxigenic *Escherichia coli* of serogroups O6 and O8. *Infect Immun* **21,** 638–647 (1978).
6. Boylan, M., Smyth, C. J. & Scott, J. R. Nucleotide sequence of the gene encoding the major subunit of CS3 fimbriae of enterotoxigenic *Escherichia coli*. *Infect Immun* **56,** 3297–3300 (1988).
7. Smyth, C. J. Two mannose-resistant haemagglutinins on enterotoxigenic *Escherichia coli* of serotype O6:K15:H16 or H-isolated from travellers' and infantile diarrhoea. *J Gen Microbiol* **128,** 2081–2096 (1982).
8. Froehlich, B. J., Karakashian, A., Sakellaris, H. & Scott, J. R. Genes for CS2 pili of enterotoxigenic *Escherichia coli* and their interchangeability with those for CS1 pili. *Infect Immun* **63,** 4849–4856 (1995).
9. Thomas, L. V., McConnell, M. M., Rowe, B. & Field, A. M. The possession of three novel coli surface antigens by enterotoxigenic *Escherichia coli* strains positive for the putative colonization factor PCF8775. *J Gen Microbiol* **131,** 2319–2326 (1985).
10. McConnell, M. M., Chart, H., Field, A. M., Hibberd, M. & Rowe, B. Characterization of a putative colonization factor (PCFO166) of enterotoxigenic *Escherichia coli* of serogroup O166. *J Gen Microbiol* **135,** 1135–1144 (1989).
11. McConnell, M. M., Hibberd, M., Field, A. M., Chart, H. & Rowe, B. Characterization of a new putative colonization factor (CS17) from a human enterotoxigenic *Escherichia coli* of serotype O114:H21 which produces only heat-labile enterotoxin. *J Infect Dis* **161,** 343–347 (1990).
12. Anantha, R. P. *et al.* Evolutionary and functional relationships of colonization factor antigen i and other class 5 adhesive fimbriae of enterotoxigenic *Escherichia coli*. *Infect Immun* **72,** 7190–7201 (2004).
13. Clark, C. A., Heuzenroeder, M. W. & Manning, P. A. Colonization factor antigen CFA/IV (PCF8775) of human enterotoxigenic *Escherichia coli*: nucleotide sequence of the CS5 determinant. *Infect Immun* **60,** 1254–1257 (1992).
14. Hibberd, M. L., McConnell, M. M., Field, A. M. & Rowe, B. The fimbriae of human enterotoxigenic *Escherichia coli* strain 334 are related to CS5 fimbriae. *J Gen Microbiol* **136,** 2449–2456 (1990).
15. Tacket, C. O., Maneval, D. R. & Levine, M. M. Purification, morphology, and genetics of a new fimbrial putative colonization factor of enterotoxigenic *Escherichia coli* O159:H4. *Infect Immun* **55,** 1063–1069 (1987).
16. Viboud, G. I., Binsztein, N. & Svennerholm, A. M. A new fimbrial putative colonization factor, PCFO20, in human enterotoxigenic *Escherichia coli*. *Infect Immun*

61, 5190–5197 (1993).
17. Valvatne, H., Sommerfelt, H., Gaastra, W., Bhan, M. K. & Grewal, H. M. Identification and characterization of CS20, a new putative colonization factor of enterotoxigenic *Escherichia coli*. *Infect Immun* **64,** 2635–2642 (1996).
18. Nada, R. A. *et al.* Discovery and phylogenetic analysis of novel members of class b enterotoxigenic *Escherichia coli* adhesive fimbriae. *J Clin Microbiol* **49,** 1403–1410 (2011).
19. Jalajakumari, M. B., Thomas, C. J., Halter, R. & Manning, P. A. Genes for biosynthesis and assembly of CS3 pili of CFA/II enterotoxigenic *Escherichia coli*: novel regulation of pilus production by bypassing an amber codon. *Mol Microbiol* **3,** 1685–1695 (1989).
20. McConnell, M. M., Thomas, L. V., Scotland, S. M. & Rowe, B. The possession of coli surface antigen CS6 by enterotoxigenic*Escherichia coli* of serogroups O25, O27, O148, and O159: a possible colonization factor? *Curr Microbiol* **14,** 51–54 (1986).
21. Svennerholm, A. M., Vidal, Y. L., Holmgren, J., McConnell, M. M. & Rowe, B. Role of PCF8775 antigen and its coli surface subcomponents for colonization, disease, and protective immunogenicity of enterotoxigenic *Escherichia coli* in rabbits. *Infect Immun* **56,** 523–528 (1988).
22. Taniguchi, T., Fujino, Y., Yamamoto, K., Miwatani, T. & Honda, T. Sequencing of the gene encoding the major pilin of pilus colonization factor antigen III (CFA/III) of human enterotoxigenic *Escherichia coli* and evidence that CFA/III is related to type IV pili. *Infect Immun* **63,** 724–728 (1995).
23. Giron, J. A., Levine, M. M. & Kaper, J. B. Longus: a long pilus ultrastructure produced by human enterotoxigenic *Escherichia coli*. *Mol Microbiol* **12,** 71–82 (1994).
24. Aubel, D., Darfeuille-Michaud, A. & Joly, B. New adhesive factor (antigen 8786) on a human enterotoxigenic *Escherichia coli* O117:H4 strain isolated in Africa. *Infect Immun* **59,** 1290–1299 (1991).
25. Pichel, M., Binsztein, N. & Viboud, G. CS22, a novel human enterotoxigenic *Escherichia coli* adhesin, is related to CS15. *Infect Immun* **68,** 3280–3285 (2000).
26. Forestier, C., Welinder, K. G., Darfeuille-Michaud, A. & Klemm, P. Afimbrial adhesin from *Escherichia coli* strain 2230: Purification, characterization and partial covalent structure. *FEMS Microbiol Lett* **40,** 47–50 (1987).
27. Knutton, S., Lloyd, D. R. & McNeish, A. S. Identification of a new fimbrial structure in enterotoxigenic *Escherichia coli* (ETEC) serotype O148:H28 which adheres to human intestinal mucosa: a potentially new human ETEC colonization factor. *Infect Immun* **55,** 86–92 (1987).
28. Heuzenroeder, M. W., Elliot, T. R., Thomas, C. J., Halter, R. & Manning, P. A. A new fimbrial type (PCFO9) on enterotoxigenic *Escherichia coli* 09:H- LT+ isolated from a case of infant diarrhea in central Australia. *FEMS Microbiol Lett* **54,** 55–60 (1990).
29. Del Canto, F. *et al.* Identification of coli surface antigen 23, a novel adhesin of enterotoxigenic *Escherichia coli*. *Infect Immun* **80,** 2791–2801 (2012).
30. Karlsson, S. L. *et al.* Development of stable *Vibrio cholerae* O1 Hikojima type vaccine strains co-expressing the Inaba and Ogawa lipopolysaccharide antigens. *PloS one* **9,** e108521 (2014).
